# Efficient production of glycyrrhetinic acid in metabolically engineered *Saccharomyces cerevisiae* via an integrated strategy

**DOI:** 10.1186/s12934-019-1138-5

**Published:** 2019-05-28

**Authors:** Caixia Wang, Xinyao Su, Mengchu Sun, Mengting Zhang, Jiajia Wu, Jianmin Xing, Ying Wang, Jianping Xue, Xia Liu, Wei Sun, Shilin Chen

**Affiliations:** 1 Key Laboratory of Beijing for Identification and Safety Evaluation of Chinese Medicine, Institute of Chinese Materia Medica, China Academy of Chinese Medical Sciences, No. 16 Nanxiaojie, Dongzhimennei Ave, Beijing, 100700 People’s Republic of China; 2 School of Life Science, Huai Bei Normal University, Huaibei, 23500 People’s Republic of China; 3 School of Chemistry, Chemical Engineering and Life Sciences, Wuhan University of Technology, Wuhan, 430070 People’s Republic of China; 4 Agilent Technologies (China) Co., Ltd., Wangjingbei Road, Chaoyang District, Beijing, 100102 China; 5 National Key Laboratory of Biochemical Engineering, Institute of Process Engineering, Chinese Academy of Sciences, Beijing, 100190 People’s Republic of China; 6 Key Laboratory of South China Agricultural Plant Molecular Analysis and Genetic Improvement, Provincial Key Laboratory of Applied Botany, South China Botanical Garden, Chinese Academy of Sciences, Guangzhou, 510650 People’s Republic of China

**Keywords:** Glycyrrhetinic acid, Triterpene, *Saccharomyces cerevisiae*, Metabolic engineering

## Abstract

**Background:**

Glycyrrhetinic acid (GA) is the most important ingredient in licorice due to its outstanding anti-inflammatory activity and wide application in the medicine and cosmetics industries. Contemporary industrial production of GA by acid hydrolysis of glycyrrhizin which was extracted from *Glycyrrhiza* plants, is not environment-friendly and devastates farmland since the *Glycyrrhiza* rhizomes grow up to 10 m underground.

**Results:**

In this study, GA was produced through metabolically engineering *Saccharomyces cerevisiae* by introducing the entire heterogeneous biosynthetic pathway of GA. Codon optimized *CYP88D6* and *CYP72A154*, combined with *β*-*AS* (β-amyrin synthase encoding gene) and the NADPH-cytochrome P450 reductase gene of *Arabidopsis thaliana* were introduced into *S. cerevisiae*. The resulting strain (Y1) produced 2.5 mg/L of β-amyrin and 14 μg/L of GA. The cytochrome *b5* from *G. uralensis* (*GuCYB5*) was identified and the introduction of this novel *GuCYB5* increased the efficiency of GA production by eightfold. The joint utilization of the *GuCYB5* gene along with 10 known MVA pathway genes from *S. cerevisiae* were overexpressed in a stable chromosome integration to achieve higher GA production. Using the combined strategy, GA concentration improved by 40-fold during batch fermentation. The production was further improved to 8.78 mg/L in fed-batch fermentation, which was increased by a factor of nearly 630.

**Conclusions:**

This study first investigated the influence of carbon flux in the upstream module and the introduction of a newly identified *GuCYB5* on GA production. The newly identified *GuCYB5* was highly effective in improving GA production. An integrated strategy including enzyme discovery, pathway optimization, and fusion protein construction was provided in improving GA production, achieving a 630 fold increase in GA production. The metabolically engineered yeast cell factories provide an alternative approach to glycyrrhetinic acid production, replacing the traditional method of plant extraction.

**Electronic supplementary material:**

The online version of this article (10.1186/s12934-019-1138-5) contains supplementary material, which is available to authorized users.

## Background

Chinese licorice, *Glycyrrhiza,* is a leguminous plant species whose roots and rhizomes have been widely used in herbal medicine and as a natural sweetener. More than 200 active ingredients were identified in *Glycyrrhiza uralensis* of which glycyrrhizin (GL) and glycyrrhetinic acid (GA) are the major bioactive triterpenoid saponins [1]. Chemically, GL is made up of one GA as aglycone and two d-glucuronic acid [2, 3], with a wide range of pharmacological activities. These include anti-inflammatory [4], immunomodulatory, and antiviral, specifically against different DNA and RNA viruses such as human immunodeficiency virus (HIV) and severe acute respiratory syndrome (SARS)-associated coronavirus [2, 3, 5]. Clinically, GL is metabolized into GA after oral administration, which then becomes the bioactive molecule in vivo [6, 7]. Indeed, GA has been widely used in both the cosmetic and pharmaceutical industry for its whitening and anti-inflammatory effect [8].

The high market price of GA, which is nearly twice that of GL, necessitates methods for direct GA production. However, chemical synthesis of GA is not feasible because of its structural complexity. The low GA content in licorice, as precursor of GL, also makes the direct extraction of GA from plants not cost effective. Currently, GA is produced by acid hydrolysis of GL, which accounts for 2–8% of the dry weight of licorice [9]. This acid consuming hydrolysis procedure is inefficient and expensive with regard to the consequent environment pollution. On the other hand, the raw materials of GL are mainly extracted from *Glycyrrhiza* plants whose cultivation is time intensive, requiring 3–4 years to reach higher GL content. Deep growth of *Glycyrrhiza* roots and rhizomes, up to 10 m, underground also results in farmland devastation and eventual desertification after plant harvest, making GA production from *Glycyrrhiza* cultivation unsustainable.

Rapid development of functional genomics has accelerated the identification of key genes involved in the biosynthesis of natural products [10–12]. This has enabled de novo production of valuable bioactive molecules in *Escherichia coli* or *S*. *cerevisiae* using synthetic biology and metabolic engineering [10, 12]. The growing list of natural products that have been synthesized in recent years includes artemisinin [13, 14], protopanaxadiol [15], ginsenosides [16, 17], ginkgolides [18], vindoline [19], tanshinones [20, 21], strictosidine [22], and gastrodin [23]. For example, the complete biosynthetic pathway of artemisinic acid was engineered and optimized in *S. cerevisiae* [13], resulting in 25 g/L of artemisinic acid production.

**Fig. 1:**
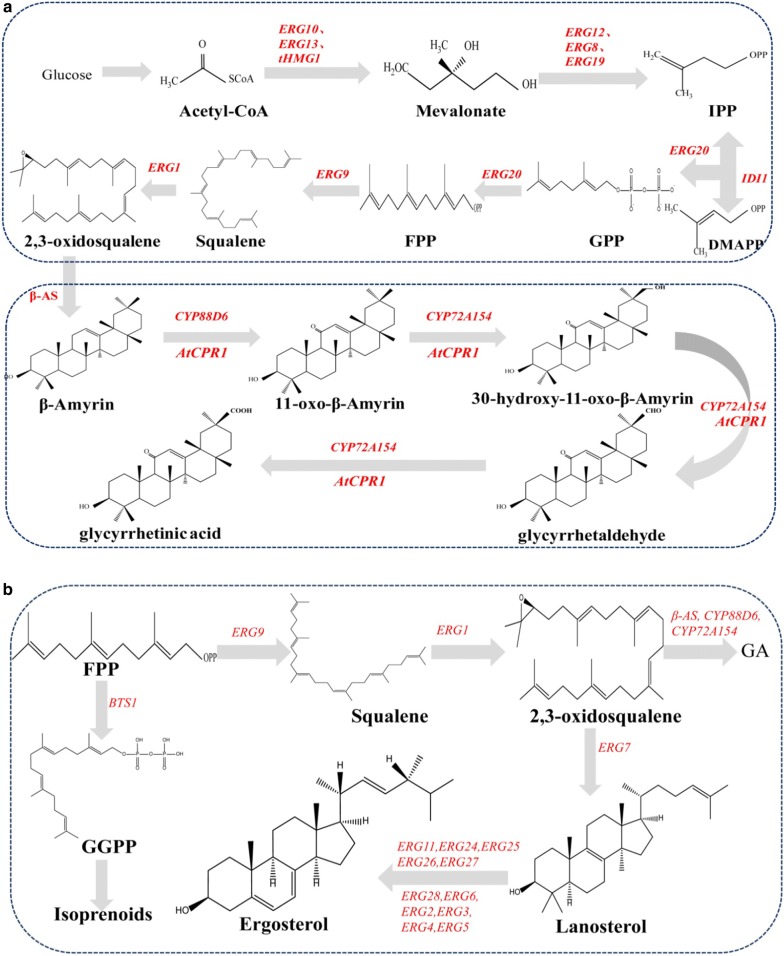
**a** Biosynthetic pathway of glycyrrhetinic acid constructed in *S. cerevisiae*. IPP: isopentenyl pyrophosphate; DMAPP: dimethylallyl pyrophosphate; GPP: geranyl diphosphate; FPP: farnesyl diphosphate; GGPP: geranylgeranyl pyrophosphate; ERG10: acetyl-CoA C-acetyltransferase; ERG13: hydroxymethylglutaryl-CoA synthase; tHMG1: truncated 3-hydroxy-3-methylglutaryl-CoA reductase 1; ERG12: mevalonate kinase; ERG8: phosphomevalonate kinase; ERG19: diphosphomevalonate decarboxylase; IDI1: dimethylallyl diphosphate isomerase; ERG20: Farnesyl pyrophosphate synthetase; ERG9: squalene synthase; ERG1: squalene epoxidase; β-AS: β-amyrin synthase; AtCPR1: cytochrome P450 reductase from *Arabidopsis thaliana*; CYP88D6: β-amyrin 11-oxidase; CYP72A154: 11-oxo-beta-amyrin 30-oxidase; **b** Biosynthetic pathway of ergosterol in *S. cerevisiae*. BTS1: arnesyltranstransferase; ERG7: lanosterol synthase; ERG11: sterol 14-demethylase; ERG24delta(14)-sterol reductase; ERG25: methylsterol monooxygenase; ERG26: sterol-4-alpha-carboxylate 3-dehydrogenase; ERG27: 3-keto-steroid reductase; ERG28: ergosterol biosynthesis 28; ERG2: C-8 sterol isomerase; ERG3: C-5 sterol desaturase; ERG4: delta(24(24(1)))-sterol reductase; ERG5: C-22 sterol desaturase; ERG6: sterol 24-C-methyltransferase

As a typical oleanane-type triterpenoid, GA is synthesized from isopentenyl diphosphate (IPP) and dimethylally diphosphate (DMAPP), two intermediates of the mevalonate pathway (MVA) that presents within the cytoplasm and mitochondria of all plant cells. The cyclization of 2, 3-oxidosqualene to β-amyrin catalyzed by β-amyrin synthase is the first reaction towards GA biosynthesis [24]. After this site-specific oxidation at positions C-11 and C-30, β-amyrin are catalyzed by two cytochrome P450 enzymes, CYP88D6 and CYP72A154, leading to the formation of GA [9, 25]. Subsequently, GA is converted to GL through a recently identified UDP-dependent glucuronosyltransferases, *GuUGAT* [26]. The complete gene set for GA biosynthesis makes GA production in yeast possible (Fig. 1a).

Considering the expanding GA demand and the limited *Glycyrrhiza* resource supply, our objective was to produce GA from glucose using engineered *S. cerevisiae*. As a precursor of GA, β-amyrin was first produced in yeast by Keasling’s group [27], with a resulting concentration of 6 mg/L. It was further improved to 138 mg/L by Li’ s group [28]. We finally improved it into 158 mg/L now (data is under review). From β-amyrin to GA, it needs two cytochrome P450 enzymes, CYP88D6 and CYP72A154, making the synthesis of GA difficult in yeast. Recently, Li’s group successfully achieved glycyrrhetinic acid synthesis in *S. cerevisiae* and boosted its concentration by introducing efficient cytochrome P450s (CYP450s: Uni25647 and CYP72A63) and pairing their reduction systems [29]. However, only influence of carbon flux in downstream module (the below box in Fig. 1a) on GA production was investigated in that study. Besides CYP450s and CPR1, the carbon flux flowing into MVA pathway from acetyl-CoA, especially into the synthesis of 2,3-oxidosqualene, is crucial to the production of GA. The pipeline of carbon flux flowing into 2,3-oxidosqualene in MVA pathway determined how high that GA’s production can reach. Thus, detailed influence of carbon flux in upstream module of GA should be investigated. Cytochrome b5 genes were believed as an another important factor that facilitates the expression of CYP450s, as it can provide an electron for P450 oxidation. For example, with the identification of *CYB5* from *Artemisia annua*, the production of artemisinic acid was improved sharply in *S. cerevisiae*.

Based on the background, we first integrated heterologously GA formation genes into the chromosome of the *S. cerevisiae* to create the GA-producing strain. Then, we investigated native upstream module factors of MVP that likely regulate GA biosynthesis. We used transcriptome mining to identify cytochrome *b5*, which was capable of facilitating GA production. Lastly, we constructed a first generation GA-producing *S. cerevisiae* (Fig. 1a). Our study provides an efficient solution for GA production in yeast, and sets a solid foundation for other triterpenoids biosynthesis using metabolically engineered yeast.

## Results

### Construction of the glycyrrhetinic acid synthetic pathway in *S. cerevisiae*

Overall, the flowchart of yeast strain construction in this study were shown in Additional file 1: Fig. S1.

We attempted to construct the metabolic pathway for GA production in *S. cerevisiae*. The four gene cassettes of *β*-*AS*, *CYP88D6*, *CYP72A154*, and *AtCPR1* were first integrated into the chromosome of *S. cerevisiae* Cen.pk2-1D at the rDNA site. Six positive colonies (Y0-1,2,3,4,5,6) were randomly selected and verified by PCR amplification. Analysis of cell extracts by GC–MS confirmed the successful production of β-amyrin and GA in these constructed strains ranging 0.02–1.35 mg/L for β-amyrin and 6.1–12.6 μg/L for GA (Additional file 1: Fig. S2A, B).

**Fig. 2:**
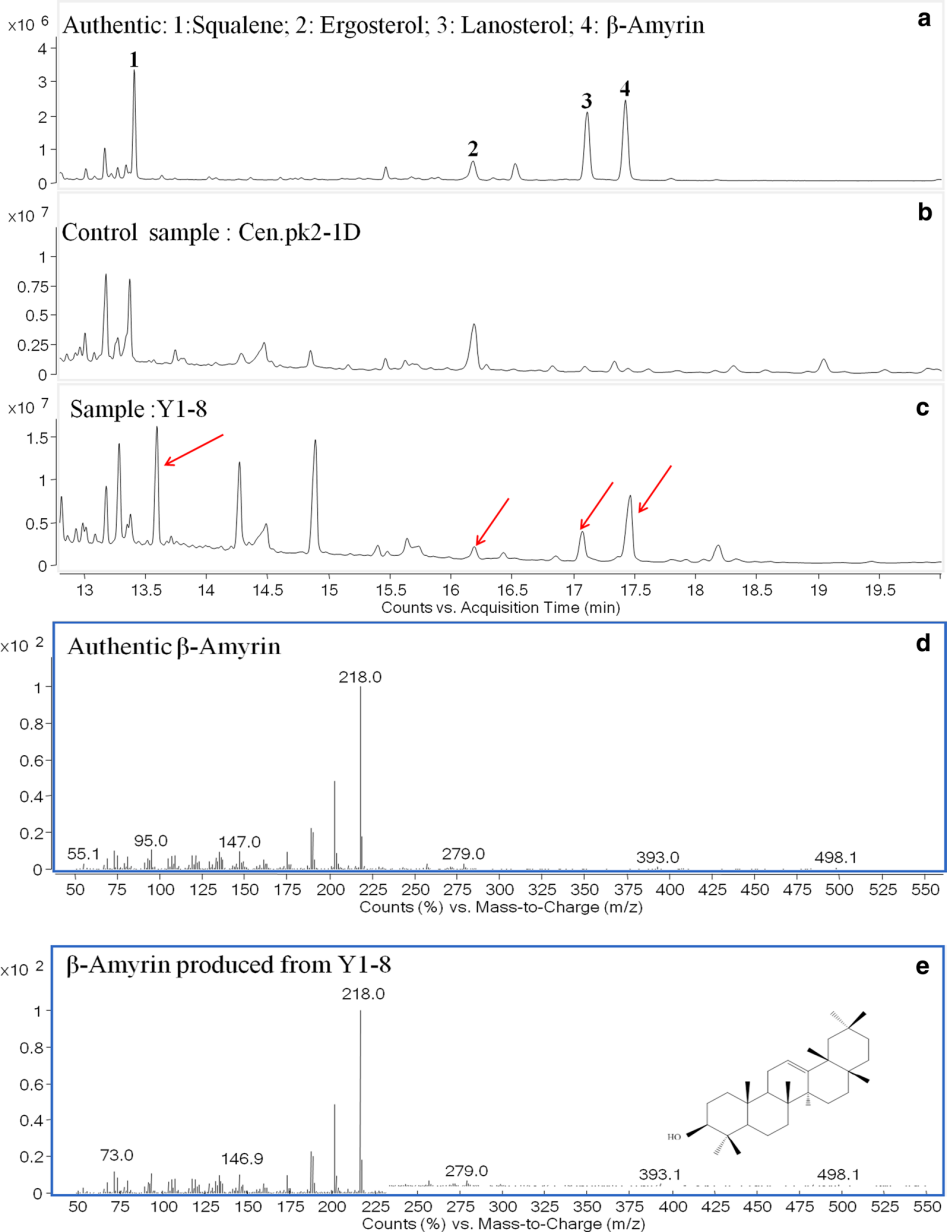
Fermentation product identification for strain Y1-8; **a** GC–MS analysis of (1) squalene, (2) ergosterol, (3) lanosterol, (4) β-amyrin standards; **b** GC–MS analysis of cell extraction of control strain Cen.pk 2-1D; **c** GC–MS analysis of cell extraction of constructed strain Y1-8; **d** mass spectra of β-amyrin standard; **e** mass spectra of β-amyrin produced from strain Y1-8

**Fig. 3:**
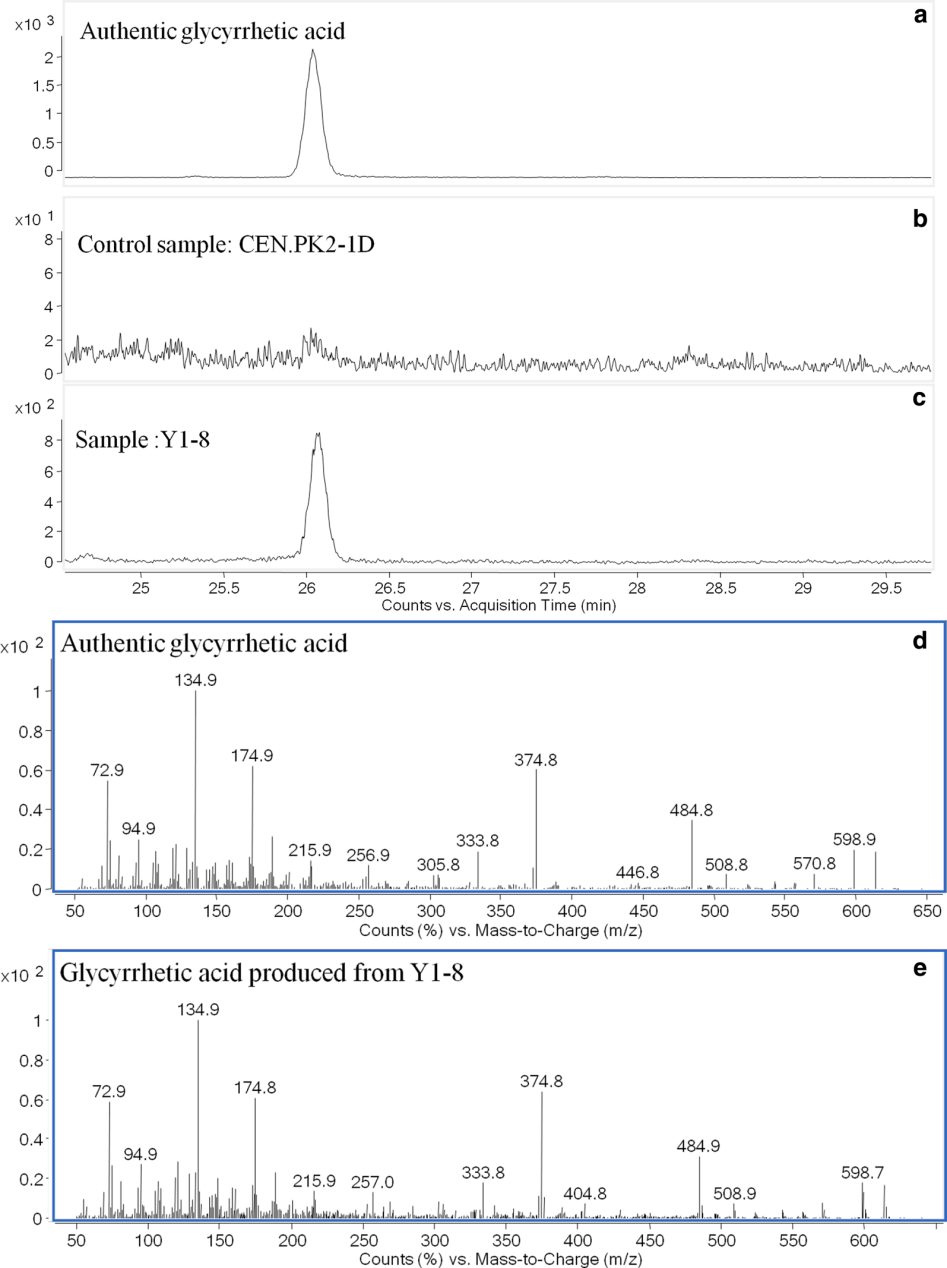
De novo glycyrrhetic acid production from strain Y1-8; **a** GC–MS analysis of glycyrrhetic acid standards; **b** GC–MS analysis of cell extraction of control strain CEN.PK2-1D; **c** GC–MS analysis of cell extraction of constructed strain Y1-8; **d** mass spectra of glycyrrhetic acid standard; **e** mass spectra of glycyrrhetic acid produced from strain Y1

**Fig. 4:**
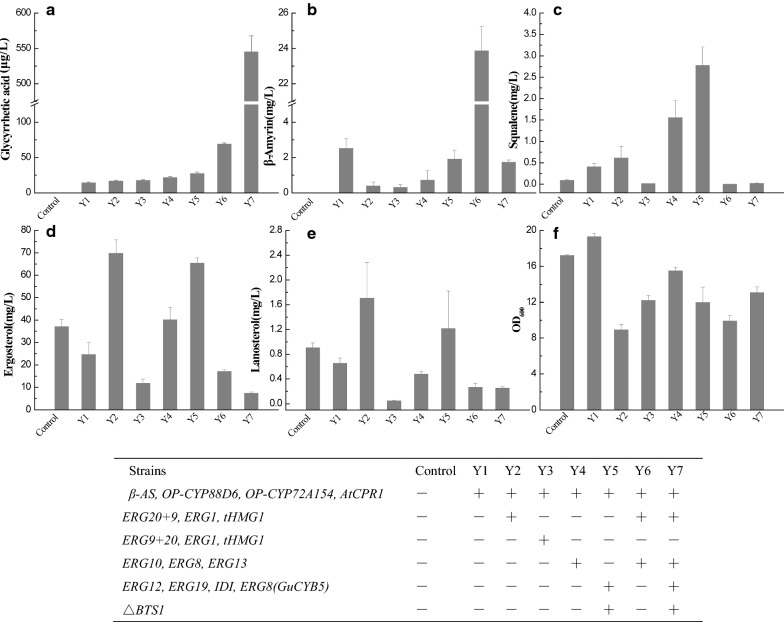
Production of glycyrrhetic acid, β-amyrin, squalene, ergosterol and lanosterol by engineered *S. cerevisiae* strains. **a** Cell growth; **b** production of glycyrrhetic acid; **c** production of β-amyrin; **d** production of squalene; **e** production of ergosterol; **f** production of lanosterol. Three repeats were performed for each strain, and error bars represent standard deviation; β-AS: β-amyrin synthase; ERG20: Farnesyl pyrophosphate synthetase; ERG9: squalene synthase; ERG1: squalene epoxidase; tHMG1: truncated 3-hydroxy-3-methylglutaryl-CoA reductase 1; AtCPR1: cytochrome P450 reductase from *Arabidopsis thaliana*, ERG10: acetyl-CoA C-acetyltransferase, ERG8: encoding phosphomevalonate kinase, ERG13: hydroxymethylglutaryl-CoA synthase; ERG12: mevalonate kinase; ERG19: diphosphomevalonate decarboxylase; IDI1: dimethylallyl diphosphate isomerase; ERG8: phosphomevalonate kinase; BTS1: arnesyltranstransferase; GuCYB5: cytochrome b5 from *G. uralensis*

We then optimized the codons of *CYP88D6* and *CYP72A154*, and integrated them together with *β*-*AS* and *AtCPR1* into *S. cerevisiae* Cen.pk2-1D. Also, six colonies (Y1-1,2,3,5,8) were selected and verified by PCR amplification. Concentrations of β-amyrin and GA of these six strains were shown in Additional file 1: Fig. S2C, D. Based on the results, strain Y1–8 (designed as Y1) gave the best performance. The highest concentrations of β-amyrin (Fig. 2) and GA (Fig. 3) produced by strain Y1 improved to 17 mg/L and 19.5 μg/L, respectively (Fig. 4a, b).

Fermentation of Y1 and the blank control *S. cerevisiae* Cen.pK2-1D was conducted in YPD media with 2% glucose as the carbon source. While the control clone cannot synthesize β-amyrin and GA, its lanosterol concentration is about 140-fold that of Y1 at approximately the same level of ergosterol but only 20% of squalene levels (Fig. 4c–e). Considering the fact that in yeast sterol (lanosterol and ergosterol) and GA share the same precursor of squalene, these results suggest that the introduction of the GA synthetic pathway in *S. cerevisiae* Cen.pk2-1D consumed the squalene pool and decreased the carbon flux into sterol.

### Overexpressing genes in MVA pathway for improving glycyrrhetinic acid production

In eukaryotic cells, farnesyl pyrophosphate (FPP), squalene and 2, 3-oxidosqualene are the three consecutive precursors of GA. As the branching point, FPP that is produced from IPP and DMAPP by *ERG20* can be converted to either GGPP by *BTS1* or squalene by *ERG9*. A previous study demonstrated that the over-expression of *BTS1* and fusion expression of *ERG20*-*BTS1* channeled the FPP flux to miltiradiene production rather than to other FPP-consuming pathways [20]. This implies that the loss of function for *BTS1* should favor the formation of squalene, thus improving GA production.

It was also reported that over-expression of a truncated HMG-CoA reductase gene (*tHMG1*) effectively improved triterpoind production [15, 16] while over-expression of the mevalonate pathway (MVP) genes in *S. cerevisiae* CEN.PK2-1D doubled artemisinic acid production [13]. Thus, we evaluated the over-expression effects of ten MVA genes on GA production.

#### Overexpression of *ERG20, ERG9, ERG*1 and *tHMG1* on the production of glycyrrhetinic acid

To channel FPP flux to squalene production, *EG20* and *ERG9* were fused in *ERG20*-*ERG9* and *ERG9*-*ERG20* two configurations, driven by constitutive TDH3 promoters and terminated by CYC1 terminators. Different configurations may have different effect on enhancement of squalene pool which was crucial for GA production. Thus, *ERG20*-*ERG9* fused gene was integrated into the delta sites of strain Y1, together with expression cassettes of *ERG1* and *tHMG1*, resulting Y2 series strains (Y2-1,2,3,4,5). Similar integration was adopted for *ERG9*-*ERG20* gene cassette, giving Y3 series strains (Y3-1,2,3,4,5). GA concentration was further analyzed for these positive colonies. It turned out that the *ERG9*-*ERG20* clone Y3-1 (designed as Y3) had the highest GA concentration but the *ERG20*-*ERG9* clone Y2-4 (designed as Y2) had higher average GA content (Additional file 1: Fig. S3), both of which were approximately 20% higher than Y1 (Fig. 4a). It’s noteworthy that different configuration of fusion resulted in distinct concentrations of squalene, ergosterol, lanosterol, all of which were higher than Y1, suggesting that the carbon source was mostly directed to sterol biosynthesis rather than GA production upon the integration of *ERG20*+*9*, *ERG1* and *tHMG1*.

#### Overexpression of *ERG10*, *ERG8*, *ERG*13, *ERG12, ERG19*, *IDI1* on the production of glycyrrhetinic acid

We further evaluated the effects of the upstream genes of the MVP pathway on GA production. *ERG10* (encoding acetyl-CoA C-acetyltransferase), *ERG8* (encoding phosphomevalonate kinase), and *ERG13* (encoding hydroxymethylglutaryl-CoA synthase) were integrated into the YDR007W site of strain Y1. As YDR007W site presents only one copy in the genome of *S. cerevisiae*, one positive colony was verified and designed as strain Y4 whose genotype can be seen in Table 2. Squalene, ergosterol and GA of strain Y4 increased 2.78, 1.66, and 1.5 fold, respectively, while lanosterol (0.48 mg/L) and β-amyrin (0.71 mg/L) concentrations were about 17% and 48% that of Y1. It seemed that unlike the *ERG20*+*9*, *ERG1* and *tHMG1* over-expression, the *ERG10*, *ERG8*, and *ERG13* cassette increased squalene and GA production without interfering sterol biosynthesis.

Similarly, gene expression cassettes of *ERG12* (encoding mevalonate kinase), *ERG19* (encoding diphosphomevalonate decarboxylase), *IDI1* (encoding isopentenyl diphosphate isomerase) and *ERG8* (encoding phosphomevalonate kinase) were inserted into the YPL069C site flanking the truncated *BTS1* (encoding farnesyltranstransferase) gene (Fig. 1b). Since *BTS1* catalyzes the conversion of FPP to GGPP, this construct, designated as strain Y5, should channel more carbon from FPP to squalene production (Fig. 1). Indeed, squalene of Y5 improved dramatically from 0.41 to 2.8 mg/L, and β-amyrin decreased from 2.5 to 1.9 mg/L (Fig. 4b, c). The final concentration of GA of Y5, being twice that of Y1 (Fig. 4a), demonstrated the effectiveness of this design.

### Integrated strategy for improved glycyrrhetinic acid production

Based on the results in “Overexpression of *ERG20, ERG9, ERG*1 and *tHMG1* on the production of glycyrrhetinic acid” and “Overexpression of *ERG10*, *ERG8*, *ERG*13, *ERG12, ERG19*, *IDI1* on the production of glycyrrhetinic acid”, we selected strain Y4 with *ERG10*, *ERG8*, and *ERG13* integration as the basis for further metabolic engineering. First, *ERG20*-*ERG9*, *ERG1,* and *tHMG1* were integrated into the delta sites of Y4. By this way, clones Y6(1–8) were obtained. Variation within GA concentration of eight random colonies demonstrated the integration of different copy numbers of focal genes (Additional file 1: Fig. S4). Of these colonies, Y6-1 (designed as Y6) yielded five-fold GA than that of Y1, with undetectable squalene as well as lower ergosterol and lanosterol concentration, suggesting that this protocol may efficiently direct carbon flux to GA production.

A recent study has demonstrated that cytochrome b5 (*CYB5*) can provide electrons to its interacting partner cytochromes P450 (P450), enhancing P450’s enzymatic activities. For example, over-expression of cytochrome b5 from *A. annua* resulted in a 3.5 fold increase of artemisinic acid and 40% increase of total sesquiterpene production [13]. In this study, we identified a *CYB5* gene of *G. uralensis* (Additional file 1: Fig. S5) and assembled *GuCYB5* using the TDH3 promoter and transformed it into the YPL069C site of the Y6 strain (designated as Y7). Not surprisingly, GA concentration of Y7 reached 545 μg/L, which was about 8 times that of Y6 and 40 fold more than Y1 (Fig. 4a). Additionally, a decrease of β-amyrin by 25% and lower concentrations of squalene and lanosterol was observed (Fig. 4b–d). Since sterol and triterpenoid share the same carbon flux from glucose to squalene, increased GA concentration of Y7 demonstrated that carbon flux was successfully channeled to triterpenoid biosynthesis rather than sterol.

We monitored the growth of these constructed yeast strains (Fig. 4f) and found that strain Y2 and Y6, both with higher GA production, grew slower than the control strain while the remaining strains showed similar growth rates. This seemed to suggest that intracellular accumulation of GA and its precursors encumbered normal growth of yeast. The apparent normal growth of Y7 under high GA concentration, on the other hand, may be attributed to the function of *GuCYB5*, as reported in previous artemisinic acid work [13].

### Scale-up fed-batch fermentation of GA production

**Fig. 5:**
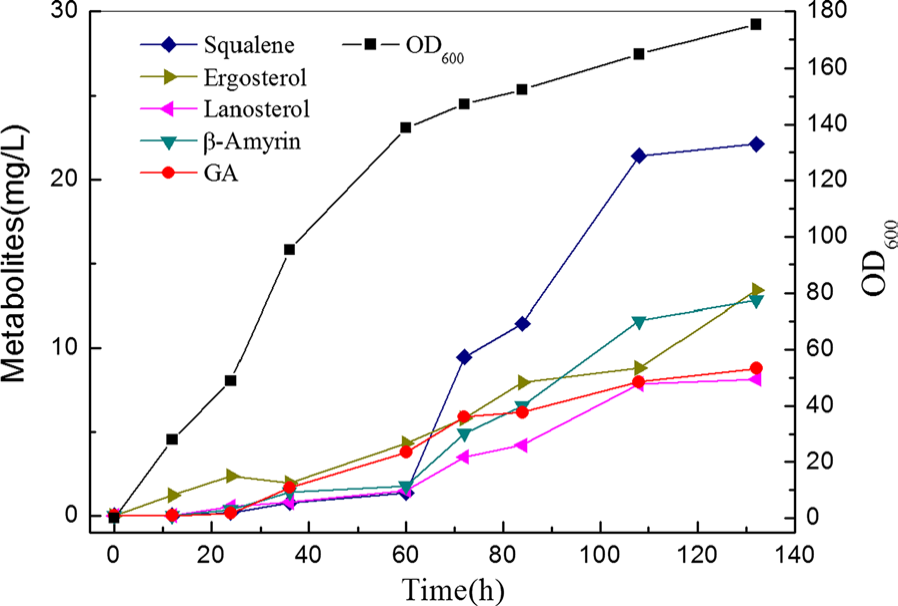
Time profile of fed-batch GA production in a 7.5 L fermentor

To further improve GA production, fed-batch fermentation was conducted in a 7.5 L fermentor. Since metabolites accumulate inside the cell, GA production is highly correlated with cell density in that higher cell density increases GA production. Thus, we monitored growth of strain Y7 on different mediums before fed-batch fermentation (Additional file 1: Fig. S6). Maximum strain growth (OD_600_ = 17.36) was obtained with the fermentation medium as expected. Fed-batch fermentation of strain Y7 in fermentation medium was conducted and results were shown in Fig. 5. Strain growth was much faster than that in the flask, as the OD_600_ was 10 times higher than that in the flask, with a maximum of 175. Final GA concentration reached 8.78 mg/L, which was nearly 630-fold higher than that produced by the original strain. The concentration of β-amyrin was 12.8 mg/L, which was a moderate level. However, the produced 11-oxo-β-amyrin, the direct GA precursor, significantly increased to 80 mg/L (data not shown). The total triterpenoid produced in the study reached to 101 mg/L. Replicate fed-batch fermentation attained GA of 6.9–7.8 mg/L. Our results illustrate an effective method for improving GA and 11-oxo-β-amyrin production.

## Discussion

Recently, much attention has been given to the synthesis of natural products utilizing microorganisms via fermentation since the process is green and sustainable [30, 31]. We selected *S. cerevisiae* for the production of plant derived terpenoids, as it provides a similar physical and physiological intracellular environment for the functional expression of cytochrome P450 (CYP450s) and uridine diphosphate glycosyltransferases (UGTs) from plants and allows for endomembrane localization and post-translational modifications.

In this study, we produced glycyrrhetinic acid using the metabolic engineering of *S. cerevisiae* by introducing the entire heterogeneous biosynthetic pathway of GA. With the overexpression of *ERG20, ERG9, ERG*1 and *tHMG1,* we were able to improve GA production by approximately 21%, although this was lower than reported in previous studies. The over expression of tHMG1 significantly increased protopanaxadiol production between 10 and 90 fold depending upon the gene copies integrated in the chromosome of *S. cerevisiae* [15]. The integration of *ERG20*, *ERG9* and *PgSQE1* (squalene epoxidase 1 encoding gene from *Panax ginseng*) increased protopanaxadiol by 3.3 times [17]. Although protopanaxadiol and GA share the same two precursors, 2 and 3-oxidosqualene, the integration of *tHMG1* did not improve GA production to the same extent as protopanaxadiol. Despite an limited increase in GA production, levels of precursors including squalene, ergosterol, and lanosterol of Y2 were much higher than Y1, specifically 50%, 188% and 161%, respectively. This suggests that the improved metabolic flux in the MVA pathway of strain Y2 by the integration of ERG20-ERG9, *ERG*1 and *tHMG1* were likely directed to the sterol synthesis, and partially to GA synthesis. This finding is supported by previous studies, which also reported the transcription level of *ERG20* and *ERG9* increased 3.3- and 2.8-fold in overexpressed *ERG20* and *ERG9* strains for β-amyrin production in the β-amyrin producing strain [28]. Our results showed a 66% increase of β-amyrin in the Y2 strain compared to strain Y1. We believe that the increase in β-amyrin through the integration of *ERG20*-*ERG9*, *tHMG1*, and *ERG1* was partially catalyzed to form both 11-oxo-β-amyrin and GA, leading to lower increases of β-amyrin. Constructing protein fusion can be highly effective in enhancing the metabolic flux channeled to the synthesis pathway of desired products by engineering their active sites to a closer proximity [20]. In this study, the fusion of ERG20 and ERG9 was constructed to direct the FPP flux to squalene production with two modules of ER20-ERG9 and ERG9-ERG20. Results indicated that the fusion of ER20-ERG9 enhanced the conversation of FPP to squalene although more carbon flux was directed into sterol.

The low yield of natural functional ingredients produced by microorganisms was mainly due to low catalytic activities of the introduced plant cytochrome P450 enzymes [32, 33]. The poor coupling between cytochrome P450 and CPR may have lead to the release of reactive oxygen species, decreasing cell growth and product yields [13, 34]. Previous findings have shown that *CYB5* facilitates and in some cases was required for the reduction of P450 enzymes by providing an electron for P450 oxidation (Additional file 1: Fig. S7) [35–37]. The introduction of newly identified *GuCYB5* in this study was highly efficient in improving GA production as indicated by our results. With AtCPR1 providing the electron for CYP450, oxidation was less efficient than CPR1 derived from *G. uralensis* (*GuCPR1*) in GA production [29]. The limited coupling between *CYP88D6*, *CYP72A154* and *AtCPR1* resulted in a relatively lower GA yield, and highlighted the effectiveness of *GuCYB5*, which significantly improved the production of GA. Therefore, future work should focus on balancing the expression between CYP88D6, CYP72A154, and CPR for heightened optimization due to the importance for cell growth and product synthesis. Formerly, *GuCPR1* was identified as presenting the highest electron transfer efficiency among several related CPRs from different plant sources recently [29]. Additionally, future work should include self-sufficient P450 reduction enzyme construction to further increase GA production efficiency.

The production level of precursors was around milligram per liter, but GA concentration was only microgram per liter in flask fermentation. In the fed-batch fermentation process, we found that 11-oxo-β-amyrin reached nearly 80 mg/L. These results demonstrate that rate-limiting steps exist in the 11-oxo-β-amyrin to GA conversion. There are two CYP450 genes responsible for catalyzing β-amyrin to GA: *CYP88D6* and *CYP72A154*, of which *CYP72A154* is a transmembrane protein (Additional file 1: Fig. S8). From this, the rate-limiting step in GA production was likely the final step catalyzed by CYP72A154 and investigating the protein engineering of CYP72A154 is imperative. We deduce that N-terminal amino acid residues used to truncate CYP72A154 will improve GA production, and further systematic investigation is needed. Like *CYP71AV1* in *A. annua*, *CYP72A154* mostly catalyzes 11-oxo-β-amyrin to form GA, leading to the intermediate formation of 30-hydroxy-11-oxo-β-amyrin and glycyrrhetaldehyde (Fig. 1a). An identification of aldehyde dehydrogenase and alcohol dehydrogenase from *G. uralensis* is also needed to improve the catalytic capacity of *CYP72A154*. Further work addressing these aspects of GA production will enhance GA production like artemisinic acid and strictosidine producing strain construction [13, 22].

Like other triterpenoids produced by yeast, GA is reconstituted in yeast at relatively lower yields, as supported by Zhu et al. [29]. It is important to mention that for the original strains, Zhu et al. used a diploid strain while we used a haploid strain making the effectiveness of strategies employed to improve GA production comparable. Specifically, Zhu et al. focused on the optimization of the CYP450 gene expression and the reduction systems where this study investigated the influence of carbon flux in the upstream module and introduction of *GuCYB5* on GA production. The genetic strategy provided by Zhu’s study combined with the workflow presented in this study would likely further improve GA production.

Here, we report a *S. cerevisiae* strain capable of producing GA, where improvement of GA production was achieved using a combined strategy comprised of enzyme discovery, pathway optimization, and fusion protein construction. A newly identified gene from *G. uralensis,* together with 10 known MVA pathway genes from *S. cerevisiae* were overexpressed in a stable chromosome integration to achieve higher GA creation. Utilizing these methods we were able to show a 40-fold increase of GA attaining 0.5 mg/L in a flask batch fermentation condition. GA production was further improved to 8.78 mg/L by fed-batch fermentation, 630 times higher than the parent strain. These results provide insights into the influence of the MVA carbon flux on β-Amyrin based triterpeniods. More importantly, we show the effectiveness of *GuCYB5* in improving GA production. In short, this study provides an effective integrated strategy for GA production using yeast, to achieve a 630 fold improvement in GA production. This strategy as well as the GA-producing strain provides solid foundation for future production of other triterpenoids in yeast.

## Conclusions

The codon optimizated *CYP88D6* and *CYP72A154*, together with the *β*-*AS* and *AtCPR1* were integrated into *S. cerevisiae*, to construct a glycyrrhetinic acid producing yeast. A novel *GuCYB5* gene, which enhances the reaction rate of *CYP88D6* and *CYP72A154*, was identified from *G. uralensis.* Over expression of different genes in the MVA pathway showed positive influences on GA production, varying from 0.21 to 5 fold increases in GA production. However, *GuCYB5* was found to be the most effective in improving GA production, specifically 8 times more effective. Through the integration of genes attributed to the MVA pathway with *GuCYB5*, GA production reached a max of 0.55 mg/L during flask fermentation, which was further improved to 8.78 mg/L in fed-batch fermentation, allowing for a 630 fold increase of the parent strain Y1. The constructed yeast strains may provide an alternative source for glycyrrhetinic acid or β-amyrin based triterpenoids and reduce the need to extract from licorice.

## Materials and methods

### Prediction and cloning of CYB5 of *G. uralensis*

By using the *CYB5* (*AtCYB5*) protein sequence from *Arabidopsis thaliana* as a reference, we were able to identify *CYB5* sequence from *G. uralensis* utilizing the website http://ngs-data-archive.psc.riken.jp/Gur/blast.pl (accessed on 2017-3-30). To clone *GuCYB5,* we used the primers found in Additional file 1: Table S1.

### Vector construction

Total RNA was isolated from the fresh roots of *G. uralensis* using the RNA prep pure Plant Kit (TIANGEN, Beijing, China). We obtained cDNA of *G. uralensis* using the Prime-Script 1st Strand cDNA Synthesis Kit (Takara, Dalian, China). To amplify *β*-*AS*, *CYPDD6*, and *CYP72A154* from the cDNA of the *G. uralensis*, we used the primers in Additional file 1: Table S1. To improve gene expression in *S. cerevisiae* we optimized the codons *CYP88D6* (OP-*CYP88D6*) and *CYP72A154* (OP-*CYP72A154*) for genes cloning into pUC57, which were synthesized by GenScript (GenScript, Nanjing, China). The optimized sequences of these are shown in the Additional file 1.

We constructed pUC19L-Ptdh3-Tcyc1, pUC19L -Ppgk1-Tadh1, pUC19L-Ptef1-Tpgk1, and pUC19L-Ptef2-Tcyc1 utilizing seamless cloning and assembly by implementing the pEASY-UniSeamless Cloning and Assembly kit (TransGen Biotech, Beijing, China) (Additional file 1: Fig. S9a). The promoters of TDH3, PGK1, TEF1, and TEF2 as well as the terminators of CYC1, PGK1, and ADH1 were amplified from the genomic DNA of *S. cerevisiae* Cen.pk2-1D with the primers shown in Additional file 1: Table S1. Primers used for seamless cloning and assembly can be seen in Additional file 1: Table S2.

The workflow for construction and seamless cloning for the following: Ptef2-*ERG19*-cyc1, Ptdh3-*CYP88D6*-Tcyc1, Ptdh3-OP-*CYP88D6*-Tcyc1, Ppgk1-*β*-*AS*-Tadh1, Ppgk1-*tHMG1*-Tadh1, Ptdh3-*ERG20*+*9*-Tcyc1, Ptdh3-ERG*9*+*20*-Tcyc1, Ptef1-*ERG1*-Tpgk1, and Ptdh3-*AtCPR1*-Tcyc1 (Additional file 1: Fig. S9b). The primers used for seamless cloning and assembly are shown in Additional file 1: Table S3.

To construct the expression cassettes for Pdth3-*ERG8*-Ttdh3 and Ptdh3-*ERG12*-Ttdh3, *ERG8* and *ERG12* genes were amplified from the genomic DNA of *S. cerevisiae* Cen.pk2-1D then digested by *Sal*I and *Not*I. After digestion they were then ligated into the *Sal*I and *Not*I digested pATP424 by T4 DNA Ligase (Takara, Dalian, China). We used the cDNA of the *G. uralensis* to amplify *GuCYB5* and ligate it into pATP424, the same as *ERG8* forming the plasmid pATP424-*GuCYB5*.

The gene expression cassettes of Padh1-*ERG10*-Tadh1, Padh1-*CYP72A154*-Tadh1, Padh1-OP-*CYP72A154*-Tadh1, and Ppgk1-*IDI1*-Tpgk1 were constructed similarly except that enzymatic digestion occurred with *Avr*II and *Fse*I rather than *Sal*I and *Not*I. Primers used for plasmid construction detection in Additional file 1: Table S4 and all plasmids used in study are shown in Table 1.

**Table 1:** Plasmids used in this study

Name	Description	Source
pUC19L	Cloning vector with multiple cloning sites, Amp	Invitrogen
pATP423	2MICRON, HIS3	Ishii et al. [38]
pATP424	2MICRON, TRP1	Ishii et al. [38]
pATP425	2MICRON, LEU2	Ishii et al. [38]
pATP406	w/o yeast origin, URA3	Ishii et al. [38]
PRS423	2MICRON, HIS3	Christianson [39]
pUC19L-*P*_*tef1*_-*T*_*pgk1*_	Cloning *P*_*tef1*_-*T*_*pgk1*_ cassette into pUC19L	This study
pUC19L-*P*_*tdh3*_-*T*_*cyc1*_	Cloning *P*_*tdh3*_-*T*_*cyc1*_ cassette into pUC19L	This study
pUC19L-*P*_*pgk1*_-*T*_*adh1*_	Cloning *P*_*pgk1*_-*T*_*adh1*_ cassette into pUC19L	This study
pUC19L-*P*_*tef2*_-*T*_*cyc1*_	Cloning *P*_*tef2*_-*T*_*cyc1*_ cassette into pUC19L	This study
pUC19L-*P*_*tdh3*_-*T*_*tdh3*_	Cloning *P*_*tdh3*_-*T*_*tdh3*_ cassette into pUC19L	This study
pUC19L-P_tef2_-ERG19-T_cyc1_	Cloning *P*_*tef2*_-*ERG19*-*T*_*cyc1*_ cassette into pUC19L	This study
pUC19L-*P*_*tdh3*_-*ERG9*+*20*-*T*_*cyc1*_	Cloning *P*_*tdh3*_-*ERG9*+*20*-*T*_*cyc1*_cassette into pUC19L	This study
pUC19L-*P*_*tdh3*_-*ERG20*+*9*-*T*_*cyc1*_	Cloning *P*_*tdh3*_-*ERG20*+*9*-*T*_*cyc1*_ cassette into pUC19L	This study
pUC19L-*P*_*tef1*_-*ERG1*-*T*_*pgk1*_	Cloning *P*_*tef1*_-*ERG1*-*T*_*pgk1*_ cassette into pUC19L	This study
pUC19L-*P*_*pgk1*_-*tHMG1*-*T*_*adh1*_	Cloning P_pgk1_-tHMG1-T_adh1_ cassette into pUC19L	This study
pUC19L-*P*_*pgk1*_-*β*-*AS*-*T*_*adh1*_	Cloning *P*_*pgk1*_-*β*-*AS*-*T*_*adh1*_ cassette into pUC19L	This study
pUC19L-*P*_*tdh3*_-*CYP88D6*-*T*_*cyc1*_	Cloning *P*_*tdh3*_-*CYP88D6*-*T*_*cyc1*_ cassette into pUC19L	This study
pUC19L-*P*_*tdh3*_-*AtCPR1*-*T*_*cyc1*_	Cloning *P*_*tdh3*_-*CPR1*-*T*_*cyc1*_ cassette into pUC19L	This study
pATP406-*ERG8*	pATP406, expressing *ERG8* gene by P_TDH3_	This study
pATP406-*ERG10*	pATP406, expressing *ERG10* gene by P_ADH1_	This study
pATP406-*ERG12*	pATP406, expressing *ERG12* gene by P_ADH1_	This study
pATP406-*ERG13*	pATP406, expressing *ERG13* gene by P_ADH1_	This study
pATP406-*IDI1*	pATP406, expressing *IDI1* gene by P_PGK1_	This study
pATP406-*CYB5*	pATP406, expressing *CY B5* gene by P_TDH3_	This study
pATP24- *CYP72A154*	PATP424, expressing *CYP72A154* gene by P_ADH1_	This study
pATP424- *AtCPR1*	pATP424, expressing *AtCPR1* gene by P_TDH3_	This study
pATP424-*β*-*AS*	pATP424, expressing *β*-*AS* gene by P_PGK1_	This study
pATP424-*OP*-*CYP72A154*	pATP424, expressing *OP*-*CYP72A154* gene by P_ADH1_	This study
pATP424-OP88D6	pATP424, expressing *OP*-*CYP88D6* gene by P_TDH3_	This study

### Strain construction

*Saccharomyces cerevisiae* Cen.pk2-1D, which was used as the parental strain, was purchased from EUROSCARF. To transform *S. cerevisiae* strains, we used the standard lithium acetate method or the electroporation method. Gene cassettes of Ppgk1-*β*-*AS*-Tadh, P tdh3-*CYP88D6*-Tcyc1, Padh1-*CYP72A154*-Tadh1, and Ptdh3-*AtCPR1*-Tcyc1 were amplified from the corresponding plasmids. Sequences of rDNA-up and rDNA-down were amplified from the DNA of *S. cerevisiae* Cen.pk2-1D using the primer set of GA-rDNA-up-F&R and GA-rDNA-down-F&R. To amplify the sequence of Leu2 marker from pRS425 we used the primer set of GA-Leu2-F&R. These six amplified DNA fragments were electroporated into *S. cerevisiae* Cen.pk2-1D using the DNA assembly method reported in previous studies [15] followed by selection on a SD-Leu plate. Similarly, fragments of Ppgk1-*β*-*AS*-Tadh, Ptdh3-OP-*CYP88D6*-Tcyc1, Padh1-OP-*CYP72A154*-Tadh1, Ptdh3-*AtCPR1*-Tcyc1, rDNA-up and rDNA-down were amplified and transferred into *S. cerevisiae* Cen.pk2-1D. We randomly selected colonies from the SD-Leu plates, after which six positive ones were verified. The highest GA-producing strain was designed as strain Y1.

Strain Y2 was constructed by integrating *ERG20*+*9* (fused gene of ERG20 and ERG9 in ERG20+ERG9 way), *tHMG1,* and *ERG1* into the delta sites of Y1. Gene expression cassettes of Ptdh3-*ERG20*+*9*-Tcyc1, Ppgk1-*tHMG1*-Tadh1, and Ptef1-*ERG1*-Tpgk1 were amplified from their corresponding plasmids. Delta-up and Delta-down were amplified from the DNA of *S. cerevisiae* Cen.pk2-1D using the primer set Delta-up-F&R and Delta-down-F&R. We amplified the Ura3 marker from pRS426 using the primer set of delta-URA-F&R. After transferring these five fragments into strain Y1 we selected five positive colonies with the highest GA-producing strain designed as strain Y2. The construction of strain Y3 was similar to strain Y2 except *ERG9*+*20* (fused gene of ERG20 and ERG9 in ERG9+ERG20 way) was used in place of *ERG20*+*9*.

By integrating *ERG10*, *ERG8*, and *ERG13* into the YDR007W site of strain Y1, we created strain Y4. We amplified gene expression cassettes of *ERG10*, *ERG8*, and *ERG13* from plasmids of pATP406-*ERG10*, pATP406-*ERG8*, and pATP406-*ERG12*. The trp-up and trp-down sequences were amplified from DNA of *S. cerevisiae* Cen.pk2-1D using the primer set of Trp-up-F&R and Trp-down-F&R. The His marker was amplified from pRS423 using the primer set of Trp-his-F&R. We electroporated these five fragments into stain Y1 and selected a positive colony from the SD-His plate, which we designed as strain Y4.

Strain Y5 was constructed by inserting *ERG12*, *ERG8*, ERG19, and *IDI1* into the YPL069C site which was the *BTS1* gene coding area. Gene expression cassettes of *ERG12*, *ERG8*, *ERG19,* and *IDI1* were amplified from plasmids of pATP406-*ERG12*, pATP406-*ERG8*, pUC19L-P_tef2_-*ERG19*-T_cyc1_, and pATP406-*IDI1* using the primer set of BTS1-E12-F&R, BTS1-E8-F&R, BTS1-E19-F&R, and BTS1-IDI1-F&R. We amplified the BTS1-up and BTS1-down sequences from DNA of *S. cerevisiae* Cen.pk2-1D using the primer set of BTS1-up-F&R and BTS1-down-F&R. We used the primer set of BTS1-Trp-F&R to amplify the Trp marker from pATP424. These six fragments were transferred into strain Y1, after selection from SD-Trp plates.

Strain Y6 was constructed by integrating *ERG20*+*9* (fused gene of ERG20 and ERG9 in ERG9+ERG20 way), *tHMG1,* and *ERG1* into the delta sites of Y4. Fragments obtained were the same strain as in Y2 construction. Positive colonies were selected from SD-leu-his-ura plates and verified through PCR amplification and sequencing.

Strain Y7 was constructed by integrating the gene cassettes of *ERG12*, *GuCYB5*, *ERG19,* and *IDI1* into the YPL069C site of strain Y6. The gene expression cassette of *GuCYB5* was amplified from pATP406-*GuCYB5* using the primer set of BTS1-Cyb5-F and BTS1-Cyb5-R. Other fragments were obtained as mentioned in strain Y5 construction.

The genomic organization of these strains can be seen in Additional file 1: Fig. S10. Primers used for integration are shown in Additional file 1: Table S5 and all strains used are shown in Table 2.

**Table 2:** Strains used in this study

Name	Description	Source
Cenpk2-1D	MATα ura3-52 trp1-289 leu2-3,112 his3Δ1 MAL2-8C SUC2	EUROSCARF
Y0	*P*_*pgk1*_-*β*-*AS*-*T*_*adh1*_, *P*_*tdh3*_-*CYP88D6*-*T*_*cyc1*_, *P*_*adh1*_-*CYP72A154*-*T*_*adh1*_ and *P*_*tdh3*_-*AtCPR1*-*T*_*cyc1*_ cassettes and LEU2 marker gene were integrated into rDNA site of Cenpk2-1D	This study This study
Y1	*P*_*pgk1*_-*β*-*AS*-*T*_*adh1*_, *P*_*tdh3*_-*OPCYP88D6*-*T*_*cyc1*_, *P*_*adh1*_-*OPCYP72A154*-*T*_*adh1*_ and *P*_*tdh3*_-*AtCPR1*-*T*_*cyc*1_ cassettes and LEU2 marker gene were integrated into rDNA site of Cenpk2-1D	This study
Y2	P_tdh3_-*E20*+*9*-*T*_*cyc1*_(*P*_*tdh3*_-*E20*+*9*-*T*_*cyc*1_), *P*_*tef1*_-*ERG1*-*T*_*pgk1*_ and *P*_*pgk1*_-*tHMG1*-*T*_*adh1*_ cassettes and URA_3_ marker gene were integrated into delta site of Y1	This study
Y4	*P*_*tdh3*_-*ERG8*-*T*_*tdh3*_, *P*_*adh1*_-*E13*-*T*_*adh1*_ and *P*_*adh1*_-*E10*-*T*_*adh1*_ cassettes and HIS3 marker gene were integrated into *TRP1* site of Y1	This study
Y5	*P*_*adh1*_-*ERG12*-*T*_*adh1*_, *P*_*tdh3*_-*Cyb5*-*T*_*tdh3*_, *P*_*tef2*_- *ERG19*-*T*_*cyc1*_ and *P*_*pgk1*_-*IDI*- *T*_*pgk1*_ cassettes and HIS3 marker gene were integrated into *BTS1* site of Y1	This study
Y6	P_tdh3_-*E20*+*9*-*T*_*cyc1*_(*P*_*tdh3*_-*E20*+*9*-*T*_*cyc*1_), *P*_*tef1*_-*ERG1*-*T*_*pgk1*_ and *P*_*pgk1*_-*tHMG1*-*T*_*adh1*_ cassettes and URA3 marker gene were integrated into delta site of Y4	This study
Y7	*P*_*adh1*_-*ERG12*-*T*_*adh1*_, *P*_*tdh3*_-*Cyb5*-*T*_*tdh3*_, *P*_*tef2*_- *ERG19*-*T*_*cyc1*_ and *P*_*pgk1*_-*IDI*- *T*_*pgk1*_ cassettes and HIS3 marker gene were integrated into *BTS1* site of Y6	This study

### Yeast cultivation and medium

To select positive colonies during engineering, we used an SD dropout medium supplemented with the corresponding dropout powder (SD-His-Leu-Trp-Ura). All strains were first pre-cultured into 15 mL culture tubes containing 2 mL medium, and grown at 30 °C, 250 rpm and OD_600_ of approximately 1.0. Flasks (50 mL) containing 15 mL medium were then inoculated to an OD_600_ of 0.05 with these seed cultures. In the batch fermentation, strains (Cen.pK2-1D, Y1–Y7) were grown in YPD medium (yeast extract 20 g/L, tryptone 10 g/L and glucose 20 g/L) at 30 °C under 250 rpm for 6 days in the flask. After 144 h cultivation, cell density and metabolites concentration of all samples were measured. Flask fermentation results are presented as the mean ± S.D. of at least three independent experiments.

The fermentation medium was composed of glucose (19.5 g/L), (NH4)_2_SO_4_ (15 g/L), KH_2_PO4 (8 g/L), MgSO_4_·7H_2_O (6.15 g/L), vitamin solution (12 mL/L), and a trace metal solution (10 mL/L). The trace metal solution contained: ZnSO_4_·7H_2_O (5.75 g/L), MnCl_2_·4H_2_O (0.32 g/L), anhydrous CuSO_4_ (0.32 g/L), CoCl_2_·6H_2_O (0.47 g/L), Na_2_MoO_4_·2H_2_O (0.48 g/L), CaCl_2_·2H_2_O (2.9 g/L), FeSO_4_·7H_2_O (2.8 g/L), 0.5 M EDTA (80 mL/L). The vitamin solution contained: biotin (0.05 g/L), calcium pantothenate (1 g/L), nicotinic acid (1 g/L), myo-inositol (25 g/L), thiamine HCl (1 g/L), pyridoxal HCl (1 g/L), and *p*-aminobenzoic acid (0.2 g/L). The medium used in fermentation was based on media described previously [15, 17].

Strain Y7 was used for the production of GA through fed-batch fermentation. Seed culture was prepared by inoculating several colonies into a 250 mL flask containing 50 mL culture medium, and incubating at 30 °C at 250 rpm for 24 h. Fed-batch fermentation was conducted in a 7.5 L bioreactor with 3 L fermentation media. The size of the inoculum was 5%. The fermentation conditions were set at 30 °C, with a pH = 6, controlled by 10 M NaOH. We intermittently added 800 g/L of glucose to maintain a 30 g/L concentration at 12, 24, 36, 48 and 72 h time points.

### Analysis

Optical densities at 600 nm (OD_600_) of all strains after 6 days fermentation were measured using a Shimadzu UV-2550 spectrophotometer.

Yeast cells after 6 days fermentation were collected by centrifugation at 10,000*g* for 5 min and crushed using Bead Beater (BioSpec, USA) after which ultrasonic extraction with 2 mL extraction solutions (acetone:methanol = 1:1) occurred 2 times. The extracts were trimethylsilylated with *N*-methyl-*N*-trimethylsilyltrifluoroacetamide (Sigma-Aldrich) at 80 °C for 30 min.

The samples were analyzed by an Agilent Technology 7890 GC, coupled with a 7000C Triple Quadrupole MS (Agilent Technologies, Santa Clara, CA USA). Two microliters of the sample was injected into splitless mode with an injector temperature of 300 °C. Two HP-5 ms columns (Agilent Technologies, Santa Clara, CA USA), 15 m * 0.25 mm i.d. * 0.25 μm film thickness, were connected by a purged ultimate union (PUU) to provide sample separation. A pneumatics control module to implement backflush during post-run procedures controlled the PUU. Helium carrier gas was held at a constant flow for two columns with the first column set at a flow rate of 1.1 mL/min and the second at a flow rate of 1.3 mL/min. The column temperature program was as follows: 1 min of 80 °C followed by heating the column to 310 °C at 20 °C/min and maintaining 310 °C for 17.5 min. During post-run, the oven temperature was set at 310 °C for 7 min, while the injector pressure was decreased to 2 psi and the pressure of the PUU increased to 55 psi to backflush the less volatile matrix components of the first column.

The MS transfer line and ion source temperatures were set to 300 °C and 280 °C, respectively. The Quadrupole temperature was Q1 = Q2 = 150 °C and solvent delay was 5 min.

For quantification, we collected data in multiple reaction monitoring (MRM) mode using squalene, lanosterol, ergosterol, β-amyrin and glycyrrhetinic acid standards (all purchased from Sigma Aldrich). Two MRM transitions (one for quantification and the other for qualification) were optimized for each metabolite and are shown in Additional file 1: Table S7.

## Additional file

**Additional file 1.** Additional figures and tables.

## Data Availability

All data generated or analyzed during this study are included in this published article and its Additional file 1.
