# Supplementary material for: Efficient production of glycyrrhetinic acid in metabolically engineered Saccharomyces cerevisiae via an integrated strategy
Source: Microb Cell Fact. 2019 May 28;18:95. doi: 10.1186/s12934-019-1138-5 (PMC6540369; doi:10.1186/s12934-019-1138-5)
Supplement: Supplementary file 1 — Additional file 1. Additional figures and tables. [file 12934_2019_1138_MOESM1_ESM.docx]

Fig.S1 Flowchart of yeast strain construction in this study, strains marked with asterisk and red indicates the selected strains used for GA production detection


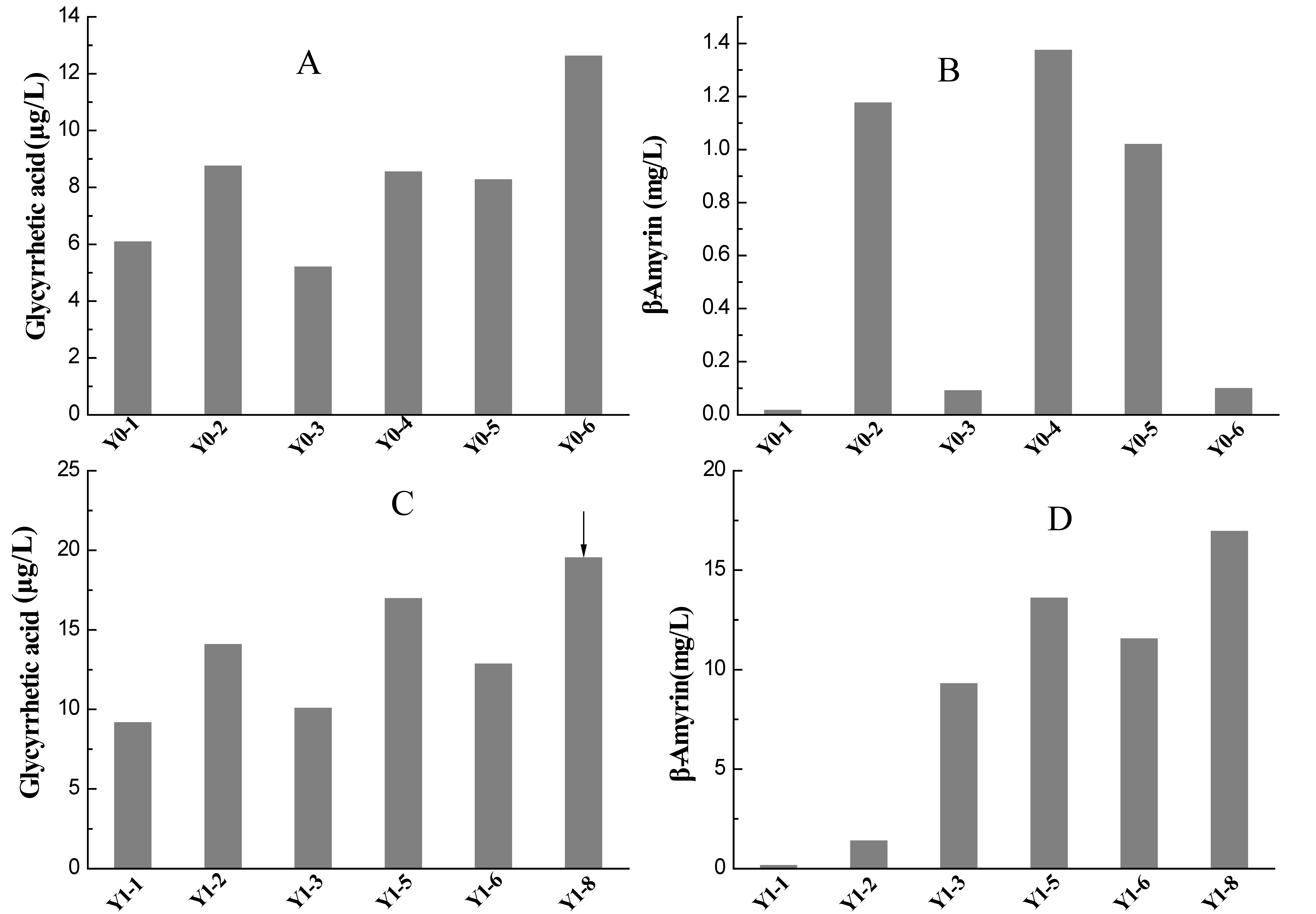


Fig.S2 Glycyrrhetinic acid and β-amyrin production of Y0 and Y1 series strains

Fig.S3 Glycyrrhetinic acid production of Y2 and Y3 series strains

Fig. S4 Glycyrrhetinic acid production of Y6 series strains


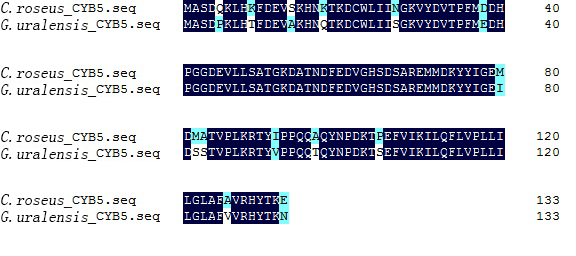


Fig.S5 Identification of *G. uralensis* cytochrome b5**.** A *G. uralensis* cytochrome b*5* cDNA sequence was identified from *G. uralensis* database (http://ngs-data-archive.psc.riken.jp/Gur/blast.pl) by searching for amino acid sequence similarity to *Artemisia annua and* *Catharanthus roseus* cytochrome b*5*. The translated *G. uralensis* cytochrome b*5* sequence was found to have 89% amino acid sequence identity with *C.roseus* cytochrome b*5*.

Fig. S6 Growth curve of strain Y7 under different media.


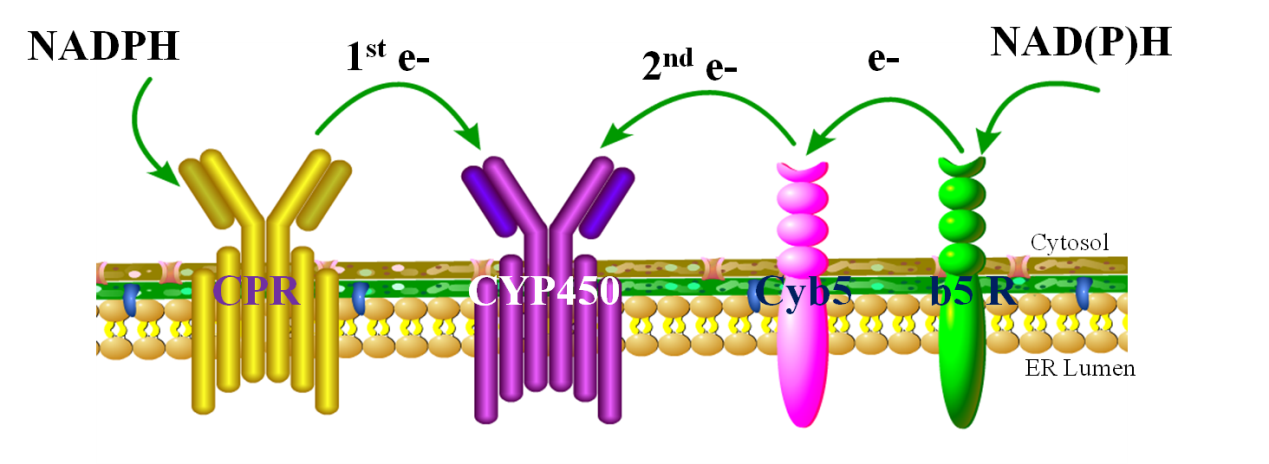


Fig.S7 A C-terminally membrane anchored cytochrome b5 (Cyb5) is required to provide the second electron for the CYP450 oxidation, Cyb5 being itself reduced by a cytrochrome b5 reductase (b5R).


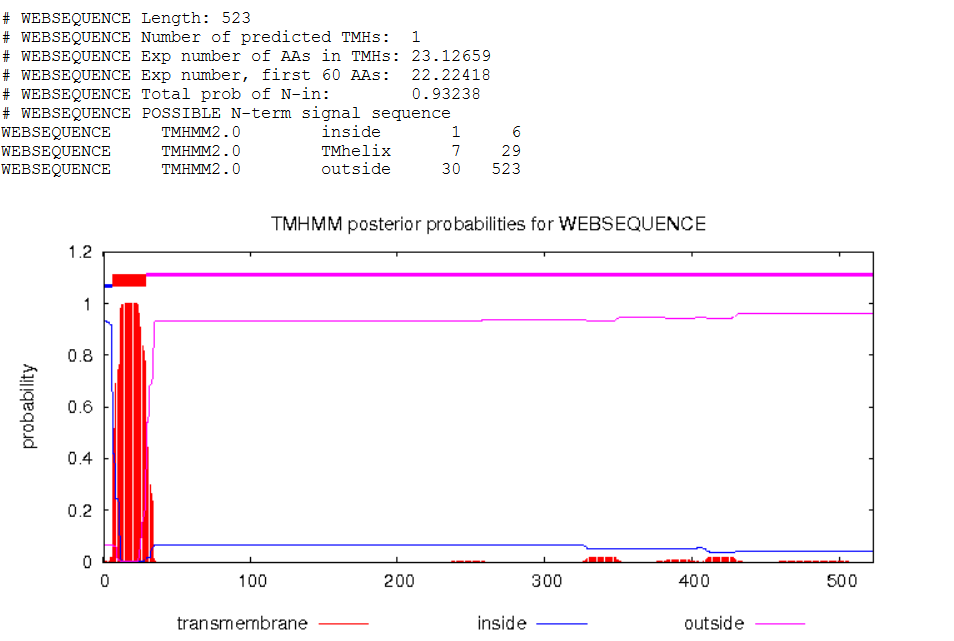


Fig.S8 Transmembrane of protein encoding by CYP72A154


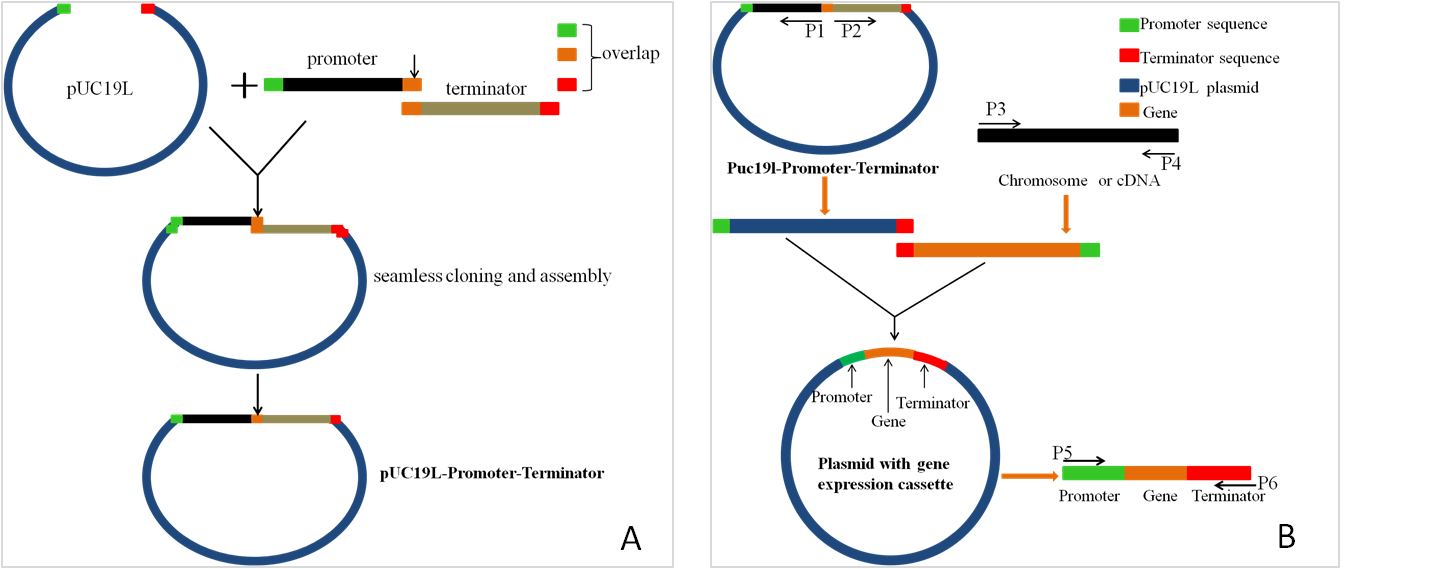


Fig. S9: A: Construction of plasmid of Promoter-Terminator cassettes; B: Construction of plasmid of gene expression cassettes

Fig. S10: Genomic organization of constructed strains in this study

Table S1 Primers used vectors ans strain construction

| Primers | Sequences |
| --- | --- |
| Xmal-β-AS-F | TCCCCCC GGGATGTGGAGGCTGAAGATAGCG |
| Ascl-β-AS-F | TTGGCGCGCCTTAAGCTGGAGTGGAAGGCAA |
| Xmal-β-AS-F | TCCCCCC GGGATGTGGAGGCTGAAGATAGCG |
| Ascl-β-AS-F | TTGGCGCGCCTTAAGCTGGAGTGGAAGGCAA |
| CYP88D6-F | ATGGAAGTACATTGGGTTTGCA |
| CYP88D6-R | CTAAGCACATGAAACCTTTATCACC |
| SalI-OPCYP88D6-F | CGCGTCGACATGGAAGTTCATTGGGTTTGTAT |
| NotI- OPCYP88D6-R | AAAAAGCGGCCGCTTATGCACAAGAAACTTTAATAACTTTA |
| AvrII-CYP72A154-F | CGCCCTAGGATGGATGCATCTTCCACACCAG |
| FseI-CYP72A154-R | AAGGGCCGGCCTTACAGTTTATGCAGAATGATGGG |
| AvrII-OP72A154-F | CGCCCTAGGATGGATGCTTCTTCAACTCCAG |
| FseI-OP72A154-R | AAGGGCCGGCCTTATAATTTATGCAAAATAATTGGTGC |
| AvrII-t-OP72A154-F | AACGCCCTAGGACTTTGTGGTTGAGACCAAAGAGA |
| AvrII-ERG9-F | AACGCCCTAGGATGGGAAAGCTATTACAATTGGC |
| FseI-ERG9-R | CCAAGGGCCGGCCTCACGCTCTGTGTAAAGTGTATATA |
| FseI-ERG20-R | CCAAGGGCCGGCCTATTTGCTTCTCTTGTAAAC |
| AvrII-E20-F | AACGCCCTAGGATGGCTTCAGAAAAAGAAAT |
| SalI-Gu- CPR1-F | CGCGTCGAC ATGCTTGCTGAGAAAGGTGGGAAG |
| NotI-Gu-CPR1-R | AGGAAAAAAGCGGCCGCTCACCATACATCACGCAAATACC |
| SalI-Gu-NADPHFR-F | CGCGTCGAC ATGGATGATGATCAATATGAGGAG |
| NotI-Gu-NADPHFR-R | AGGAAAAAAGCGGCCGCTCACCAGACATCCCTGAGGTAA |
| SalI- CPR1-F | CGCGTCGAC ATGACTTCTGCTTTGTATGCTTCCG |
| NotI- CPR1-R | AAAAAGCGGCCGC TCACCAGACATCTCTGAGGTATCTT |
| SalI-GuCYB5-F | CGCGTCGACATGGCTTCAGATCCAAAGCTTC |
| tHMG-F | TGCAGACCAATTGGTGAAAACT |
| tHMG-R | TTAGGATTTAATGCAGGTGACG |
| P-PGK1-F | AAAGATGCCGATTTGGGC |
| P-PGK1-R | GTTTTATATTTGTTGTAAAAAGTAGATAAT |
| P-TDH3-F | GACACAAGGCAATTGACCCAC |
| T-TDH3-R | TTTGTTTGTTTATGTGTGTTTATTC |
| P-TEF2-F | GATAGGTCAAGATCAATGTAAACAATTAC |
| P-TEF2-R | AAACGTTTAGTTAATTATAGTTCGTTGA |
| T-CYC1-F | GTTTAAACACAGGCCCCTTTTC |
| T-CYC1-R | AAAATATGCACATGAGGCGAA |
| T-PGK1-F | GAAATAAATTGAATTGAATTGAAATC |
| T-PGK1-R | AGCTTTAACGAACGCAGAATTTTC |
| T-ADH1F | GCGAATTTCTTATGATTTATG |
| T-ADH1R | GAATGACGATGAAGATAGAGC |
| P-TEF1-F | TCAATAGTCATACAACAGAAAGC |
| P-TEF1-R | TTTGTAATTAAAACTTAGATTAGAT |
| ERG20-R | GAACAAAGTTTACAAGAGAAGCAAATAG |
| ERG9-R | TATATACACTTTACACAGAGCGTGA |
| ERG1-R | TTGTTTGGTGAGTTGATTGGTTAA |
| Leu2ORF-F | ATGTCTGCCCCTATGTCTGCC |
| Leu2ORF-R | TTAAGCAAGGATTTTCTTAACTTCTT |
| P-ADH1-F | AAGGAAAAAAGCGGCCGCGTTGTCCTCTGAGGACATAAAATACA |
| P-ADH1-R | CCGGAATTCCCCATACATCGGGATTCCTATAATA |
| P-TRP-F | GCGTGTTTATGCTTAAATGCGTA |
| URA3-ORF-F | ATGTCGAAAGCTACATATAAG |
| URA3-ORF-R | TTAGTTTTGCTGGCCGCATC |
| HIS-ORF- F | ATGACAGAGCAGAAAGCCCTA |
| HIS-ORF-R | TTAGTTTTGCTGGCCGCATC |

Table S2 Primers used for pUC19L-promotors-termintors construction

| Primers | Sequences |
| --- | --- |
| QZpUC19L_FW-1 | CATATTTTGGCATGCAAGCTTGGCGT |
| QZpUC19L_RV-1 | GACCTATCGTACCGAGCTCGAATT |
| QTEF2_FW-1 | AGCTCGGTACGATAGGTCAAGATCAATGTAA |
| QTEF2_RV-1 | CTGTGTTTAAACGTTTAGTTAATTATAGTTCG |
| ZCYC1_FW-1 | CTAAACGTTTAAACACAGGCCCCTTTTCCTTTG |
| ZCYC1_RV-1 | TGCATGCCAAAATATGCACATGAGGC |
| QZpUC19L_FW-2 | GCTTTTATGGCATGCAAGCTTGGCGT |
| QZpUC19L_RV-2 | AGGAATCTGTACCGAGCTCGAATT |
| QPGK1_FW-2 | CTCGGTACAGATTCCTGACTTCAACT |
| QPGK1_RV-2 | TCGCGTTTAAACTGTTTTATATTTGTTGTAAAA |
| ZADH1_FW-2 | AACAGTTTAAACGCGAATTTCTTATGATTT |
| ZADH1_RV-2 | TGCATGCCATAAAAGCGATATAACGTCTT |
| QZpUC19L_FW-3 | CATATTTTGGCATGCAAGCTTGGCGT |
| QZpUC19L_RV-3 | TTCAACGGGTACCGAGCTCGAATT |
| QTDH3_FW-3 | CTCGGTACCCGTTGAAAAGAACTTAC |
| QTDH3_RV-3 | CTGTGTTTAAACTTTGTTTGTTTATGTGTGT |
| ZCYC1_FW-3 | CAAAGTTTAAACACAGGCCCCTTTTCCTTT |
| ZCYC1_RV-3 | TGCATGCCAAAATATGCACATGAGGC |
| QZpUC19L_FW-4 | TCCAAGCTGGCATGCAAGCTTGGCGT |
| QZpUC19L_RV-4 | ACTATTGAGTACCGAGCTCGAATT |
| QTEF1_FW-4 | CTCGGTACTCAATAGTCATACAACAGAAAG |
| QTEF1_RV-4 | CAATGTTTAAACTTTGTAATTAAAACTTAGATTAG |
| ZTPGK1_FW-4 | CAAAGTTTAAACATTGAATTGAATTGAAATC |
| ZTPGK1_RV-4 | TGCATGCCAGCTTGGAGCAGGAAGAAT |

Table S3 primers used for gene expression cassettes construction

| Primers | Sequences |
| --- | --- |
| TEF1_pUC19L_E1_FW | TTGGTTAAATTGAATTGAATTGAAATCGAT |
| TEF1_pUC19L_E1_RV | GCAGACATTTTGTAATTAAAACTTAGATTAGATTG |
| E1_FW_ TEF1 | ATTACAAAATGTCTGCTGTTAACGT |
| E1_RV_ TEF1 | AATTCAATTTAACCAATCAACTCACCA |
| TEF2_pUC19L_E9_FW | GAGCGTGAACAGGCCCCTTTTCCTTTG |
| TEF2_pUC19L_E9_RV | TTTCCCATGTTTAGTTAATTATAGTTCGTTGA |
| E9_FW | AACTAAACATGGGAAAGCTATTACAATTG |
| E9_RV | GGGCCTGTTCACGCTCTGTGTAAAG |
| TDH3_pUC19L_E20-9_FW | GAGCGTGAACAGGCCCCTTTTCCTTTG |
| TDH3_pUC19L_E20-9_RV | TCTGAAGCCATTTTGTTTGTTTATGTGTGTTT |
| E20-E9_FW | CAAACAAAATGGCTTCAGAAAAAGAAA |
| E20-E9_RV | GGGCCTGTTCACGCTCTGTGTAAAG |
| TDH3_pUC19L_E9-20_FW | GCAAATAGGTTTAAACACAGGCCC |
| TDH3_pUC19L_E9-20_RV | TTTCCCATTTTGTTTGTTTATGTGTGTTT |
| E9-E20_FW | CAAACAAAATGGGAAAGCTATTACAATTG |
| E9-E20_RV | GTTTAAACCTATTTGCTTCTCTTGTAAAC |
| TDH3_pUC19L_CPR1- FW | TCTGGTGAACAGGCCCCTTTTCCTTTG |
| TDH3_pUC19L_CPR1- RV | GCAGAAGTCATTTTGTTTGTTTATGTGTGTTT |
| CPR1_FW | CAAACAAAATGACTTCTGCTTTGTATG |
| CPR1_RV | GGGCCTGTTCACCAGACATCTCTGAGGT |
| PGK_pUC19L -AS- FW | TTTATGTCGACCTCGAGGGCATGCAAGCTTGG |
| PGK _pUC19 L -AS- RV | GAATCTGATATCGTACCGAGCTCGAATTC |
| β-AS_FW-PUC19 | CTCGGTACGATATCAGATTCCTGACTTCAACTCA |
| β-AS_RV-PUC19 | TGCATGCCCTCGAGGTCGACATAAAAGCGATATAACGT |
| TDH3-PUC19-op88d6_FW | GTGCATAAAAACACAGGCCCCTTTTCC |
| TDHPUC19 -op88d6_RV | ACTTCCATAAACTTTGTTTGTTTATGTGT |
| Op88D6_FW | CAAAGTTTATGGAAGTTCATTGGGTTTG |
| Op88D6_RV | CTGTGTTTTTATGCACAAGAAACTTTAATAAC |
| TEF2-PUC19L-E19_FW | AGGAATAAGTTTAAACACAGGCCCCTTTTC |
| TEF2-PUC19L-E19_RV | ACGGTCATGTTTAGTTAATTATAGTTCGTTGA |
| ERG19_FW | AACTAAACATGACCGTTTACACAGCATCCGTT |
| ERG19_RV | GTTTAAACTTATTCCTTTGGTAGACCAGTCT |
| AvrII-E10-F | AACGCCCTAGGATGTCTCAGAACGTTTACATTG |
| FseI-ERG10-R | CCAAGGGCCGGCCTCATATCTTTTCAATGACAATAGAGGAA |
| AvrII-E12-F | AACGCCCTAGGATGTCATTACCGTTCTTAACTTCTGC |
| FseI-ERG12-R | CCAAGGGCCGGCCTTATGAAGTCCATGGTAAATTCG |
| SalI-ERG8-F | CGCGTCGACATGTCAGAGTTGAGAGCCTTCAG |
| NotI-ERG8-R | AAAAAGCGGCCGCTTATTTATCAAGATAAGTTTCCGGAT |
| AvrII-E13-F | AACGCCCTAGGATGAAACTCTCAACTAAACTTTGTTG |
| FseI-E13-R | CCAAGGGCCGGCCTTATTTTTTAACATCGTAAGATCTTCT |
| XmaI-IDI-F | TCCCCCCGGGATGACTGCCGACAACAATAGTATG |
| AscI- IDI-R | TTGGCGCGCCTTATAGCATTCTATGAATTTGCCTG |

Tables S4 primers used for plasmid construction detection

| pATP-F1 | GTTGTCCTCTGAGGACATAAAATACA |
| --- | --- |
| pATP-R1 | AGCTTTAACGAACGCAGAATTTTC |
| pATP-F2 | GGAGCGATTTGCAGGCATTT |
| pATP-R2 | TCGTCATTGTTCTCGTTCCC |
| pATP-F3 | ACAGGGGCACAAACAGGCAA |
| pATP-R3 | GCGCATCAAGAAAAAACACA |
| pATP-F4 | CCAAGGGGGTGGTTTAGTTT |
| pATP-R4 | TGTTCGGGTTCAGCGTATTT |
| pATP-F2 | GGAGCGATTTGCAGGCATTT |
| pATP-R2 | TCGTCATTGTTCTCGTTCCC |
| pATP-F3 | ACAGGGGCACAAACAGGCAA |
| pATP-R3 | GCGCATCAAGAAAAAACACA |
| R-PUC19L-F | ACGACGGCCAGTGAATTCGAG |
| R-PUC19L-R | CAGCTATGACCATGATTACGCCA |

Table S5 Primers used for PCR amplification of gene expression cassettes for integration

| Primers | Sequences |
| --- | --- |
| GA-rDNA-down-F | GAACTGGGTTACCCGGGGCACCTGTC |
| GA-rDNA-down-R | CCTTACCTGAGCTTCAAGACGTTATATCGCTTTTATTTTCCTCTAATCAGGTTCCACCA |
| GA-ADH1-F | GGGGTATCTGTTTGGTGGAACCTGATTAGAGGAAAATAAAAGCGATATAACGTCTTGAA |
| GA-PGK-R | ATATTCGTTTTTTTCAGGTAAGTTCTTTTCAACGGAGATTCCTGACTTCAACTCAAG |
| GA-TDH3-F | AATATCTGTGCGTCTTGAGTTGAAGTCAGGAATCTCCGTTGAAAAGAACTTACCTGAA |
| GA-CYC1-R | GTATTATAGGAATCCCGATGTATGGGGTTTGTGCTAAAATATGCACATGAGGCGAACAA |
| GA-ADH1p-F | GATTTCTGACTTTGTTCGCCTCATGTGCATATTTTAGCACAAACCCCATACATCGGGAT |
| GA-ADH1t-R | GATTTCTGACTTTGTTCGCCTCATGTGCATATTTTGTTGTCCTCTGAGGACATAAAATA |
| GA-CYC1-F | CTCGGTGTGTATTTTATGTCCTCAGAGGACAACAAAATATGCACATGAGGCGAACAAAG |
| GA-TDH3-R | AGAATAGACCGAGATAGGGTTGAGTGTTGTTCCACCGTTGAAAAGAACTTACCTGAA |
| GA-Leu2-F | ATATTCGTTTTTTTCAGGTAAGTTCTTTTCAACGGTGGAACAACACTCAACCCTATCTC |
| GA-Leu2-R | AAACAACCTTTAGACTTACGTTTGCTACTCTCATTTATGGGAAATGCTTCAAGAAGGTA |
| GA-rDNA-up-F | TTAAGTCAATACCTTCTTGAAGCATTTCCCATAAATGAGAGTAGCAAACGTAAGTCTAA |
| GA-rDNA-up-R: | CTCACTATT TTT TAC TGCGGAAGCGG |
| Delta-down-F: | TCGAGGAGAACTTCTAGTATATTC |
| Delta-down-R | CCCTTACCTGAGCTTCAAGACGTTATATCGCTTTTATTGGAAGCTGAAACGTCTAACGG |
| delta Adh1-F | CACACAAATCAAGATCCGTTAGACGTTTCAGCTTCCAATAAAAGCGATATAACGTCTTG |
| delta PGK-R | AAAGTTGGGTGGTCGCTTTCTGTTGTATGACTATTGAAGATTCCTGACTTCAACTCAAG |
| delta-TEF1-F | ATAATATCTGTGCGTCTTGAGTTGAAGTCAGGAATCTTCAATAGTCATACAACAGAAAG |
| delta-TPGK-R | TTGATTTCTGACTTTGTTCGCCTCATGTGCATATTTTAGCTTGGAGCAGGAAGAATACA |
| delta- CYC1F | TTAGATCCAGTATAGTGTATTCTTCCTGCTCCAAGCTAAAATATGCACATGAGGCGAAC |
| delta-TDH3-R | AAAAAGAATAAAAAAAAAATGATGAATTGAATTGAAACCGTTGAAAAGAACTTACCTGA |
| delta- URA-F | ATATATTCGTTTTTTTCAGGTAAGTTCTTTTCAACGGTTTCAATTCAATTCATCATTTT |
| delta-URA-R | ACTAGTTAGTAGATGATAGTTGATTTCTATTCCAACAGGGTAATAACTGATATAATTAA |
| delta-up-F | AAATTAGAGCTTCAATTTAATTATATCAGTTATTACCCTGTTGGAATAGAAATCAACTA |
| delta-up-R | GGATATAGGAATCCTCAAAATG |
| T-Delta-Ptdh3-F | ATAATATCTGTGCGTCTTGAGTTGAAGTCAGGAATCTAAGAATACGTAAATAATTAATA |
| T-Delta-Ttdh3-R | GGTATTATAGGAATCCCGATGTATGGGGTTTGTGCTTCCTGGCGGAAAAAATTCATTTG |
| T-Delta-Padh1-F | TAAAGTTTACAAATGAATTTTTTCCGCCAGGAAGCACAAACCCCATACATCGGGATTC |
| T-Delta-PGK-R | ATATTCGTTTTTTTCAGGTAAGTTCTTTTCAACGGAGATTCCTGACTTCAACTCAAGAC |
| T-Delta-adh1-r | CGCCTCATGTGCATATTTTGTTGTCCTCTGAGGACATAAAATACACACCGAGAT |
| T-Delta -CYCI-F | CTCGGTGTGTATTTTATGTCCTCAGAGGACAACAAAATATGCACATGAGGCGAAC |
| Trp-up-R | CACGACTCATCTCCATGCAGTTGG |
| Trp-up-F | CCAACGATGTTCCCTCCACCAAAGGTGTTCTTATGTAGCTTATGGCATGTCTGGCGATG |
| Trp-His-R | GTTTGAAAAGTTTTATCATCGCCAGACATGCCATAAGCTACATAAGAACACCTTTGGTG |
| Trp-His-F | GGTATTATAGGAATCCCGATGTATGGGGTTTGTGCTTCGCGCGTTTCGGTGATGACGGTG |
| Trp-E10-R | GTCAGAGGTTTTCACCGTCATCACCGAAACGCGCGAAGCACAAACCCCATACATCGGGA |
| Trp-E10-F | AGGAAAATCACTACTATTAATTATTTACGTATTCTTGTTGTCCTCTGAGGACATAAAA |
| Trp-E8-R | ATCTCGGTGTGTATTTTATGTCCTCAGAGGACAACAAGAATACGTAAATAATTAATAGT |
| Trp-E8-F | ATCTCGGTGTGTATTTTATGTCCTCAGAGGACAACTCCTGGCGGAAAAAATTCATTTG |
| Trp-E13-R | TTTTAAAGTTTACAAATGAATTTTTTCCGCCAGGAGTTGTCCTCTGAGGACATAAA |
| Trp-E13-F | TTTACTATTATCTTCTACGCTGACAGTAATATCAAAGCACAAACCCCATACATCGGG |
| Trp-down-R | GGTATTATAGGAATCCCGATGTATGGGGTTTGTGCTTTGATATTACTGTCAGCGTAGAAG |
| Trp-down-F | GCAAGAATACCAAGAGTTCCTCGGT |
| BTS1-UP-R | CTGAAAAGGCCGCCTGTTTTAT |
| BTS1-UP-F | AAATAGCAAATTTCGTCAAAAATGCTAAGAAATAGCAGCATAGCAGAAATTACGTGTTT |
| BTS1-TRP-R | AACATATGCAAAAACACGTAATTTCTGCTATGCTGCTATTTCTTAGCATTTTTGACGA |
| BTS1-TRP-F | GGTATTATAGGAATCCCGATGTATGGGGTTTGTGCTGCGTGTTTATGCTTAAATGCGTA |
| BTS1-E12-R | AGACGCATATAAGTACGCATTTAAGCATAAACACGCAGCACAAACCCCATACATCGGGA |
| BTS1-E12-F | TAGGAAAATCACTACTATTAATTATTTACGTATTCTTGTTGTCCTCTGAGGACATAAAAT |
| BTS1-Cyb5-R | ATCTCGGTGTGTATTTTATGTCCTCAGAGGACAACAAGAATACGTAAATAATTAATAG |
| BTS1-Cyb5-F | AACAAAGTAATTGTTTACATTGATCTTGACCTATCTCCTGGCGGAAAAAATTCATTTG |
| BTS1-E19-R | TTTTAAAGTTTACAAATGAATTTTTTCCGCCAGGAGATAGGTCAAGATCAATGTAAACA |
| BTS1-E19-F | GTTTAATAACTCGAAAATTCTGCGTTCGTTAAAGCTAAAATATGCACATGAGGCGAACA |
| BTS1-IDI-R | TGATTTCTGACTTTGTTCGCCTCATGTGCATATTTTAGCTTTAACGAACGCAGAATTTTC |
| BTS1-IDI-F | TTGTTATATATAATAGACCACTTATCCGAATTGTGAAAAGATGCCGATTTGGGCGCGAA |
| BTS1-down-R | CCAAAATAAAGGATTCGCGCCCAAATCGGCATCTTTTCACAATTCGGATAAGTGGTCTA |
| BTS1-down-F | ATAAAACAGGCGGCCTTTTCAGA |

Table S6 target masses of metabolics

| Compound | Retention  time | Quantified  transition | Collision  Energy(eV) | Qualitative  mass | Collision Energy(eV) |
| --- | --- | --- | --- | --- | --- |
| Squalene | 13.373 | 136.9->95.1 | 5 | 136.9 -> 81.0 | 5 |
| ergosterol | 16.118 | 336.8->69.1 | 25 | 336.8 -> 119.1 | 25 |
| lanosterol | 17.031 | 497.9 -> 393.5 | 10 | 483.0 -> 393.5 | 5 |
| β-Amyrin | 17.34 | 217.9 -> 203.2 | 5 | 189.9 -> 175.2 | 10 |
| Enoxolone | 25.876 | 374.8 -> 135.1 | 15 | 374.8 -> 257.3 | 15 |

*OP-CYP88D6*:

ATGGAAGTTCATTGGGTTTGTATGTCAGCTGCAACTTTGTTGGTTTGTTACATCTTCGGTTCTAAGTTCGTTAGA

AATTTGAACGGTTGGTACTACGATGTTAAGTTGAGAAGAAAGGAACATCCATTACCACCAGGTGACATGGGTTGG

CCATTAATTGGTGACTTGTTGTCTTTTATTAAGGATTTCTCTTCAGGTCATCCAGATTCTTTTATTAACAATTTG

GTTTTGAAGTACGGTAGATCAGGTATCTATAAGACTCATTTGTTCGGTAACCCATCTATCATCGTTTGTGAACCA

CAAATGTGTAGAAGAGTTTTGACAGATGATGTTAACTTCAAGTTGGGTTACCCAAAGTCTATTAAAGAATTGGCT

AGATGTAGACCAATGATTGATGTTTCAAACGCAGAACATAGATTGTTTAGAAGATTAATCACATCTCCAATCGTT

GGTCATAAAGCTTTAGCAATGTACTTGGAAAGATTGGAAGAAATCGTTATTAATTCATTGGAAGAATTGTCTTCA

ATGAAGCATCCAGTTGAATTGTTGAAGGAAATGAAGAAAGTTTCTTTTAAAGCTATCGTTCATGTTTTTATGGGT

TCTTCAAACCAAGATATTATTAAGAAAATTGGTTCTTCTTTTACTGATTTGTACAACGGCATGTTCTCTATCCCA

ATTAATGTTCCAGGTTTTACATTCCATAAAGCTTTGGAAGCAAGAAAGAAATTGGCTAAGATCGTTCAACCAGTT

GTTGATGAAAGAAGATTAATGATCGAAAATGGTCCACAAGAAGGTTCACAAAGAAAGGATTTGATCGATATCTTG

TTAGAAGTTAAAGATGAAAATGGTAGAAAATTAGAAGATGAAGATATTTCTGATTTGTTAATTGGTTTGTTATTT

GCTGGTCATGAATCTACTGCAACATCATTGATGTGGTCTATCACTTACTTAACACAACATCCACATATCTTGAAG

AAAGCTAAGGAAGAACAAGAAGAAATCACTAGAACAAGATTTTCTTCACAAAAACAATTGTCATTGAAGGAAATT

AAACAAATGGTTTATTTGTCTCAAGTTATTGATGAAACTTTGAGATGTGCAAACATTGCTTTTGCAACTTTTAGA

GAAGCTACAGCAGATGTTAACATCAACGGTTACATCATCCCAAAAGGTTGGAGAGTTTTGATTTGGGCTAGAGCA

ATCCATATGGATTCAGAATACTACCCAAATCCAGAAGAGTTTAATCCATCTAGATGGGATGATTACAATGCTAAA

GCAGGTACATTTTTACCATTTGGTGCTGGTTCAAGATTGTGTCCAGGTGCTGATTTGGCAAAGTTGGAAATCTCT

ATTTTCTTGCATTACTTTTTGTTAAATTACAGATTGGAAAGAATTAATCCAGAATGTCATGTTACTTCATTACCA

GTTTCTAAACCAACAGATAACTGTTTGGCTAAAGTTATTAAAGTTTCTTGTGCATAA

*OP-CYP72A154*:

ATGGATGCTTCTTCAACTCCAGGTGCAATTTGGGTTGTTTTGACAGTTATTTTAGCTGCAATTCCAATTTGGGCT

TGTCATATGGTTAACACTTTGTGGTTGAGACCAAAGAGATTGGAAAGACATTTGAGAGCACAAGGTTTGCATGGT

GACCCATACAAGTTGTCTTTGGATAACTCAAAGCAAACATACATGTTGAAGTTGCAACAAGAAGCTCAATCTAAG

TCAATCGGTTTGTCTAAGGATGATGCTGCACCAAGAATTTTCTCTTTGGCACATCAAACTGTTCATAAGTACGGT

AAAAATTCTTTTGCTTGGGAAGGTACTGCACCAAAAGTTATTATTACAGATCCAGAACAAATTAAAGAAGTTTTT

AATAAGATTCAAGATTTCCCAAAGCCAAAGTTGAACCCAATTGCTAAGTACATCTCAATCGGTTTGATCCAATAC

GAAGGTGACAAGTGGGCTAAGCATAGAAAGATTATTAACCCAGCATTCCATTTGGAAAAGTTGAAGGGCATGTTG

CCAGCTTTTTCTCATTCATGTCATGAAATGATCTCTAAGTGGAAGGGTTTGTTGTCTTCAGATGGTACATGTGAA

GTTGATGTTTGGCCATTTTTGCAAAATTTGACTTGTGATGTTATCTCAAGAACAGCTTTTGGTTCTTCATACGCT

GAAGGTGCAAAGATCTTCGAATTGTTGAAGAGACAAGGTTACGCATTGATGACTGCTAGATACGCAAGAATTCCA

TTATGGTGGTTGTTGCCATCTACTACAAAGAGAAGAATGAAGGAAATCGATAGAGGTATCAGAGATTCATTGGAA

GGTATCATCAGAAAGAGAGAAAAGGCTTTGAAGTCTGGTAAATCAACAGATGATGATTTGTTAGGTATCTTGTTG

CAATCTAACCATATCGAAAATAAGGGTGACGAAAACTCTAAATCAGCTGGTATGACTACACAAGAAGTTATGGAA

GAGTGTAAGTTGTTTTATTTGGCTGGTCAAGAAACTACAGCTGCATTGTTAGCATGGACTATGGTTTTGTTAGGT

AAACATCCAGAATGGCAAGCTAGAGCAAAGCAAGAAGTTTTGCAAATCTTCGGTAACCAAAACCCAAACTTCGAA

GGTTTGGGTAGATTGAAGATCGTTACTATGATCTTGTACGAAGTTTTGAGATTGTACCCACCAGGTATATATTTG

ACAAGAGCTTTGAGAAAGGATTTGAAGTTGGGTAATTTGTTATTGCCAGCAGGTGTTCAATTTTCTGTTCCAATC

TTGTTGATCCATCATGATGAAGGTATTTGGGGTAATGATGCTAAAGAATTCAATCCAGAAAGATTTGCAGATGGT

ATTGCTAAGGCAACAAAGGGTCAAGTTTGTTACTTCCCATTTGGTTGGGGTCCAAGAATCTGTGTTGGTCAAAAC

TTCGCTTTGTTGGAAGCAAAGATTGTTTTGTCTTTGTTGTTGCAAAACTTCTCTTTCGAATTATCACCAACTTAC
